# Perinatal depression in a cohort study on Iranian women

**Published:** 2010

**Authors:** Gholam Reza Kheirabadi, Mohamad Reza Maracy

**Affiliations:** a Assistant Professor of Psychiatry, Behavioral Sciences Research Center, Isfahan University of Medical Sciences, Isfahan, Iran; b Assistant Professor of Statistics and Epidemiology, Department of Social Medicine, Isfahan University of Medical Sciences, Isfahan, Iran

**Keywords:** Prenatal, Perinatal, Postpartum, Postnatal, Depression, Pregnancy

## Abstract

**BACKGROUND:**

Childbearing years in the women’s life are associated with the highest risk of depression. In this study depression in third trimester of pregnancy and after delivery was studied. Depressive symptom score and the proportion of mothers above a threshold were compared to indicate probable depressive disorder at each stage.

**METHODS:**

This prospective cohort study was conducted in rural areas of Isfahan province of Iran from September 2007 to January 2008. Subjects were all in their third trimester and followed up from the beginning of the study to 6-8 weeks postpartum. At all, 2156 pregnant women completed the self report questionnaires but 258 were excluded because they were incomplete and final analysis was done with 1898 samples. At the final stage the sample size was decreased to 1291.

**RESULTS:**

The prevalence of depression based on BDI score greater than 20 in last trimester of pregnancy, was 22.8% and rate of depression based on EPD score greater than 12 between 6 to 8 weeks after delivery, was 26.3%. Incidence of Post Partum Depression (PPD) in 6 to 8 weeks after delivery in those who were not clinically depressed during pregnancy was 20.1%. Results showed that history of depression, unplanned pregnancy, being housewife and having 3 or more children had significant relation with ante partum depression.

**CONCLUSIONS:**

Two main risk factors for post partum depression are previous history of depression and depression during current pregnancy. It is important to assess these variables during pregnancy in order to facilitate timely identification of women at risk.

Women in their childbearing ages are at higher risk of depression compared with any other time in their life.^1^ Longitudinal studies have shown that not only pregnancy does not protect women against the development of new onset major depression or relapse of existing major depressive illness, but also pregnancy and motherhood are periods of increased vulnerability to psychiatric disorders.^2^

Bennett et al reported a depression prevalence, about 7.4%, 12.8% and 12% during the first, second and third trimesters.^3^ Josefsson et al studied depression during late pregnancy and reported prevalence of 17% that has been decreased to 13% by 6 to 8 weeks after delivery.^4^ Evans et al in a longitudinal study followed a group of women through pregnancy to the postpartum period and reported that prevalence of depression during pregnancy was nearly as the same as the results that were reported during the postpartum period.^1^

In a meta-analysis by Gavin et al on major depression alone (major and minor depression), the combined point prevalence estimate ranged from 1.0-5.6% (6.5% to 12.9%) at different trimesters of pregnancy and months in the first postpartum year, the combined period prevalence shows that as many as 7.1% (19.2%) of women have a major depressive episode (depressive episode) during the first 3 months postpartum; most of these episodes have onset following delivery.^5^

Despite relative neglect of depression during pregnancy, studies on antenatal psychopathology have mostly assessed antenatal mood as a predictor of postnatal depression.^6^–^8^ Watson et al reported 23% of postnatal depressions were started during pregnancy.^9^ Depression during pregnancy has been associated with poor prenatal care, substance abuse, low birth weight, and preterm delivery.^10^,^11^ Psychiatric symptoms during pregnancy have physiological impacts on the fetus, which may explain some of these problems.^12^

Studies have shown that even when pregnant women who are at risk for depression are identified, they receive few treatments.^13^ Among the first researchers, who pointed out continuous process of depression through pregnancy into the postpartum period, were Gotlib et al and despite their observations, research during the past 25 years has focused on postpartum depression, so pregnancy-related depression has changed to a secondary issue.^14^ The DSM-IV-TR in postpartum-onset mood disorders diagnostic criteria, does not refer to depression during pregnancy.^15^

Depression during pregnancy has changed to an important issue during the last 4 years in the medical literature. This may be due to increase in psychiatric referrals of depressed pregnant women, patients’ increasing willingness to report symptoms to their primary caregivers or an increased awareness among physicians about the importance of depression during pregnancy for fetus development. This fact that undiagnosed and untreated depression in pregnant women persists throughout pregnancy and the postpartum period, has been caught widespread attention today.^1^ ^4^,^14^,^16^ In this study depression in third trimester of pregnancy and after delivery was studied using prospectively gathered data from a cohort of 1898 women. Depressive symptom score and the proportion of mothers above a threshold were compared to indicate probable depressive disorder at each stage.

## Methods

This prospective cohort study was conducted in rural areas of Isfahan province (with varied geographical, cultural and socio-economical properties) in central part of Iran. The health centers in the rural areas are responsible for providing health care services. They are supported by the Vice-Chancellor for Health of Isfahan University of Medical Sciences.

Resident healthcare workers (Behvarz) in the village health centers (Health Homes) provide widespread primary health care. They also assist the visiting team of general practitioners (GP) and mental health professionals to conduct periodic visits in rural areas.

The primary health care program covers all residents of the villages in Iran. The data collection system in Health Home is based on uncomputerized records of all family characteristics including pregnant and child bearing women.

The Isfahan University of Medical Sciences ethics committee approved the design of the study.

In this study all villages were selected from whole area, for full coverage of different geographical and socio-economical participants.

Participants were all rural pregnant women in Isfahan province who were in their third trimester of pregnancy. Subjects were followed up from the beginning of the study to 6-8 weeks postpartum. About 3000 eligible pregnant women were recruited in this study based on primary information from the Vice-Chancellor for Health of Isfahan University of Medical Sciences.

Of them, 425 women were excluded because of literacy problems and the rest invited to participate; 2156 pregnant women agreed and completed the self administrator questionnaires. Of all, 258 questionnaires were excluded because they were incomplete and final analysis was done on 1898 samples. The sample size in the final stage of assessment (6-8 weeks postpartum) decreased to 1291 women.

This cohort study was carried out jointly between the Behavioral Sciences Research Center (BSRC) and The Vice-Chancellor for Health of Isfahan University of Medical Sciences. Mental health professionals in local departments of the Vice-Chancellor for Health of Isfahan University supervised the data collection as well as training the healthcare workers (Behvarz) in all aspects of the study including its objectives and design. Health-care workers contacted all eligible women and requested them to attend at the Health Home at determined dates to complete the self-report questionnaires. Participant who were unable to attend, were assessed at their home. The completed questionnaires were gathered and then referred to mental health professionals in local departments by health-care workers. Mental health workers followed up women who were found to be severely depressed or suicidal at the assessment to ascertain the need for further management. The first questionnaire covered socio-demographic information (age, number of children, occupation, history of antidepressant use, attitude to pregnancy and past history of abortion) and the other ones were Persian version of Beck Depressive Inventory-II (BDI-II) and Edinburgh Postnatal Depression Scale (EPDS).

Depression during third trimester of pregnancy and 6-8 weeks postpartum was assessed using the Persian version of Beck Depression Inventory (BDI-II)^17^ and Persian version of Edinburgh Postnatal Depression Scale (EPDS).^18^ BDI is a 21-item scale with a four-point scale that ranges from 0 to 3 score; it is a self-administered scale that takes 5 to 10 minutes to complete. Depression severity of each respondent, is the sum of the score on each item, ranging from 0 to 63 score. The average internal-consistency of the total scores is 0.86 for psychiatric patients and 0.81 for normal adults. The average correlation of the BDI total scores with clinical ratings of depression was greater than 0.90 for the both psychiatric patients and normal adults. Based on the obtained score, the severity of possible depression was assigned as follow: 0-9 (no depression), 10-16 (mild depression), 17-29 (moderate depression) and 30-63 (sever depression). Cut-off for depression screening in the general population is plus 21 for clinical depression score.^19^,^20^ The EPDS is a 10-items scale focuses on the cognitive and affective features of depression rather than somatic symptoms. It is the only self-report scale that has been validated for use in pregnancy and postnatal period.^21^ This scale itself cannot confirm a diagnosis of depression; however, a score above 12 is widely used to indicate probable depressive disorder. Validation of the EPDS showed that all women with diagnosis of major depression on interview had scored above 12 on the scale. Use of this threshold gave an overall sensitivity of 86% and specificity of 78% for all forms of depression.^22^

### 

#### Data Analysis

Data were analyzed using SPSS v.15. To demonstrate the initial results, univariate odds ratios (ORs) with 95% confidence intervals (CIs) were conducted for demographic variables and psychosocial risk factors of perinatal depression. A multiple logistic regression analysis was executed to detect prenatal and postpartum depression as the dependent variables and risk factors such as number of children, occupation, history of depression, attitude to preg-nancy, mother’s age, PICCA severity, history of abortion, neonatal hospital admissions and mother’s refer to psychiatrist as independent variables based on the ORs with 95% confidence intervals (CIs).

## Results

**Figure 1:**
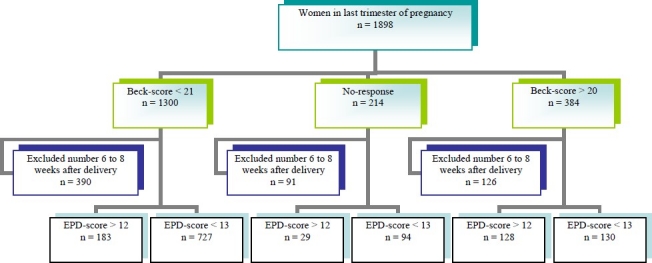
Overview of data collected from a total of 1898 available antenatal women in last trimester of pregnancy. EPDS were taken from 6 to 8 weeks after delivery in 1291 postnatal.

Of 1898 eligible pregnant women 1684 (88.7%) completed BDI-II during pregnancy and also 1291 (68%) of the total eligible pregnant women completed a postnatal EPDS included 1168 of them completed BDI-II plus 123 of noncompleted BDI-II. Point prevalence of depressed pregnant women (clinical depression) based on BDI score greater than 20 in last tri-mester of pregnancy was 22.8% (384/1684). However, postnatal response rate of depression based on EPD score greater than 12 between 6 to 8 weeks after delivery was 26.3% (340/1291). Incidence of (Post Partum Depression) PPD based on EPDS using cutoff > 12 between 6 to 8 weeks after delivery in those who were not clinically depressed during pregnancy (true definition of postpartum depression based on DSM-IV-TR) was 20.1% (183/910). Prevalence of depression based on EPDS > 12 between 6 to 8 weeks after delivery in those that were clinically depressed during pregnancy based on BDI > 20 was, 49.6% (128/258) and 51.4% of them recovered from depression after delivery (Figure 1).

The binary outcomes were BDI score and EPDS (Yes versus No) using cut-off > 20 and > 12, respectively. Logistic regression models were conducted for each explanatory variables and univariate odds ratios (ORs) with 95% confidence intervals (CIs) with respect of Beckscore in last trimester of pregnancy and EPDS in 6-8 weeks after delivery (Table 1). Some variables were categorized or reduced coding to obtain simple ORs.

**Table 1:** Univariate odds ratios (ORs) with 95% confidence intervals (CIs) of depression for variables in respect of Beck-score in last trimester of pregnancy and Edinburgh Postnatal Depression score(EPDS) in 6-8 weeks after delivery

Variable	n*	Beck-score > 20	OR (95% CIs)†	n*	EPD-score > 12	OR (95% CIs)†
Number of children:	1679			1286		
0		184.882	1.00		176.657	1.00
1		121.529	1.25 (0.87-1.46)		108.418	0.95 (0.72-1.26)
2		49.193	1.29 (0.90-1.85)		39.151	0.95 (0.64-1.42)
≥ 3		27.75	2.13 (1.30-3.51)**		17.60	1.08 (0.60-1.94)
Occupation:	1683			1290		
Employee		10.75	1.00		10.54	1.00
Housewife		373.1608	1.96 (1.00-3.86)**		329.1236	1.60 (0.79-3.21)
History of depression:	1678			1284		
No		322.1506	1.00		289.1154	1.00
Don’t know		7.25	1.43 (0.59-3.45)		7.21	1.50 (0.60-3.74)
Yes		53.147	2.07 (1.45-2.97)**		43.112	1.87 (1.25-2.79)**
Attitude to pregnancy:	1682			1290		
Desired		281.1362	1.00		264.1044	1.00
Desired but not at that time		58.188	1.92 (1.31-2.83)**		41.137	1.34 (0.87-2.06)
Undesired		44.132	1.72 (1.23-2.40)**		34.109	1.26 (0.85-1.87)
PICCA severity:	1669			1281		
No		94.450	1.00		79.335	1.00
Mediate		95.480	0.93 (0.68-1.27)		94.373	1.09 (0.77-1.54)
Moderate		122.489	1.26 (0.93-1.71)		114.389	1.34 (0.96-1.87)
Severe		67.250	1.39 (0.97-1.99)		50.184	1.21 (0.80-1.82)
Mother’s age:	1667			1274		
15-19		41.180	1.04 (0.66-1.65)		47.136	1.73 (1.07-2.81)**
20-24		158.654	1.13 (0.80-1.59		118.489	1.04 (0.71-1.54)
25-29		106.505	0.94 (0.65-1.35)		106.392	1.22 (0.82-1.81)
30-34		56.254	1.00		46.197	1.00
≥ 35		22.74	1.49 (0.84-2.67)		19.60	1.52 (0.81-2.87)
History of abortion:	1682			1290		
No		321.1434	1.00		291.1097	1.00
Yes		61.248	1.13 (0.83-1.55)		49.193	1.06 (0.75-1.51)
Neonate hospital admissions:	1229			1246		
No		241.1085	1.00		289.1102	1.00
Yes		35.144	1.12 (0.75-1.69)		42.144	1.16 (0.79-1.70)
Mother’s refer to psychiatrist: (postpartum)	1269			1279		
No		266.1236	1.00		322.1247	1.00
Yes		14.33	2.69 (1.33-5.43)**		16.32	2.87 (1.42-5.81)**

* Responses number

† Odds Ratios (95% confidence intervals)

** Significant Odds Ratio (p value < 0.05)

Table 2 shows proportion and univariate odds ratios (ORs) with 95% confidence intervals (CIs) of depression for variables with respect of EPDS > 12 with score above or below the BDI cutoff at screening in 6-8 weeks after delivery.

**Table 2:** Proportion and univariate odds ratios (ORs) with 95% confidence intervals (CIs) of depression for variables in respect of Edinburgh Postnatal Depression score (EPDS) > 12 with Beckscore < 21 or Beck-score > 20 in 6-8 weeks after delivery

Variable	n*	Beck-score < 21 EPD-score > 12 OR (95% CIs)†		n*	Beck-score > 20 EPD-score > 12 OR (95% CIs)†	
Number of children:	910			255		
0		111.491	1.00		55.119	1.00
1		51.280	0.76 (0.53-1.10)		43.84	1.22 (0.70-2.13)
2		15.100	0.60 (0.34-1.09)		19.34	1.47 (0.69-3.17)
≥ 3		6.39	0.62 (0.25-1.52)		11.18	1.83 (0.66-5.04)
Occupation:	910			257		
Employee		8.43	1.00		1.5	1.00
Housewife		175.867	1.11 (0.50-2.43)		126.252	4.00 (0.44-36.1)
History of depression:	908			257		
No		159.831	1.00		104.214	1.00
Don’t know		2.12	0.84 (0.18-3.89)		3.5	1.21 (0.18-8.12)
Yes		21.65	2.02 (1.17-3.49)**		21.38	0.76 (0.38-1.53)
Attitude to pregnancy:	910			257		
Desired		152.753	1.00		89.191	1.00
Desired but not that time		19.88	0.83 (0.44-1.59)		20.36	1.43 (0.70-2.93)
Undesired		12.69	1.09 (0.64-1.86)		18.30	1.72 (0.78-3.76)
PICCA severity:	905			255		
No		43.236	1.00		30.67	1.00
Mediate		50.273	1.01 (0.64-1.58)		37.69	1.43 (0.73-2.80)
Moderate		65.276	1.38 (0.90-2.13)		38.72	1.39 (0.71-2.69)
Severe		23.120	1.06 (0.61-1.87)		23.45	1.18 (0.56-2.49)
Mother’s age:	897			258		
15-19		29.99	2.03 (1.09-3.79)**		16.32	1.05 (0.42-2.65)
20-24		71.360	1.21 (0.72-2.03)		41.96	0.78 (0.38-1.63)
25-29		52.267	1.19 (0.69-2.04)		41.72	1.39 (0.64-2.99)
30-34		23.136	1.00		20.41	1.00
≥ 35		6.35	1.02 (0.38-2.73)		10.17	1.50 (0.48-4.71)
History of abortion:	910			257		
No		158.771	1.00		108.219	1.00
Yes		25.139	1.17 (0.74-1.87)		20.38	1.14 (0.57-2.28)
Neonate hospital admissions:	877			252		
No		154.779	1.00		110.221	1.00
Yes		23.98	1.24 (0.76-2.05)		16.31	1.08 (0.51-2.28)
Mother’s refer to psychiatrist: (postpartum)	904			255		
No		173.887	1.00		122.243	1.00
Yes		9.904	4.64 (1.77-12.2)**		6.12	0.99 (0.31-3.16)

* Responses number

† Odds Ratios (95% confidence intervals)

** Significant Odds Ratio (p value < 0.05)

Multiple logistic regressions were conducted for variables that were included in tables 1 and 2 separately. The stepwise section process yielded a model with variables such as child number, occupation, history of depression, attitude to pregnancy, and mother’s age using Beck-score in last trimester of pregnancy (Table 3). The results revealed that some vari-ables such as history of depression, pregnant mother less than 25 years of age, being house wife and undesired pregnancy were important risk factors for perinatal depression.

**Table 3:** Adjusted odds ratios (ORs) with 95% confidence interval (CIs) from multiple logistic regression model based on Beck-score>20 in last trimester of pregnancy (n = 1654)

Risk factor	OR (95%CIs)	P value
Child number:		
0	1	
1	1.24 (.91-1.69)	0.168
2	1.47 (.90-2.40)	0.119
≥ 3	2.35 (1.19-4.63)	0.014
Occupation:		
Employee	1	
Housewife	2.28 (1.10-4.73)	0.027
History of depression:		
No	1	
Yes	1.83 (1.28-2.60)	0.001
Attitude to pregnancy:		
Desired	1	
Desired but not that time	1.57 (1.02-2.41)	0.039
Undesired	1.62 (1.14-2.31)	0.007
Mother’s age:		
> 34	1.05 (.55-2.00)	0.890
15-19	1.49 (.85-2.61)	0.169
20-24	1.53 (.98-2.39)	0.061
25-29	1.10 (.73-1.65)	0.648
30-34	1	

There were 1143 postnatal EPDS scores that yielded a model with variables such as Beck score, history of depression, and mother’s age using EPDS in 6-8 weeks after delivery (Table 4).

**Table 4:** Adjusted odds ratios (ORs) with 95% confidence interval (CIs) from multiple logistic regression model based on Edinburgh Postnatal Depression (EPD) score > 12 in 6-8 weeks after delivery (n = 1143)

Risk factor	OR (95%CIs)	P value
Beck-score:		
< 21	1	
> 20	3.84 (2.84-5.18)	< 0.001
History of depression:		
No	1	
Yes	1.70 (1.11-2.60)	0.014
Mother’s age:		
> 34	1.18 (.57-2.45)	0.657
15-19	1.65 (.98-2.81)	0.062
20-24	1.04 (.68-1.59)	0.864
25-29	1.23 (.79-1.91)	0.357
30-34	1	

## Discussion

It was found that postnatal depression (during 6-8 weeks after delivery) had higher prevalence, than prenatal depression (during third trimester). However these data may suggest that depression is more likely after childbirth than pregnancy, on the other hand this difference may be related to different depression assessment scale or time table of depression assessment during pregnancy and after delivery in this study.

There is prominent evidence against this concept that pregnancy protects patients from mental illness. Prevalence of depression during pregnancy estimated from 7-15% in developed countries^1^,^23^ to 19-25% in economically underdeveloped countries.^2^,^23^ However depression prevalence is about 10% during the postpartum period and 7% after perinatal period.^1^ Evidence based studies suggest that PPD may be a part of a continuous process, with onset during pregnancy.^14^,^16^,^24^ Although some of the present results are in coordination with these findings but postpartum depression incidence of 20.1% in those that were not depressed during the last trimester of pregnancy and recovery of 51.4% depressed pregnant women after delivery, are noticeable findings which suggest that PPD and depression during pregnancy may be separate entities.

Results of this study revealed that depression has a high prevalence during pregnancy and postpartum periods and about 50% of women that were depressed during pregnancy also remained depressed after delivery but the other 50% recovered from depression that shows controversial nature of relationship between prenatal and postnatal depression. In regard to studied variables, there were no significant differences between those remained depressed after delivery and those recovered from depression.

Main risk factors for depression in pregnancy included past history of depression before pregnancy, ante partum or postpartum depression and family history of depression, especially during pregnancy or postpartum.^2^,^4^,^25^–^28^ Poverty, lack of education, and sex inequality are another predisposing factors for depression during pregnancy in all cultures.^2^,^23^,^29^–^31^

Based on a multiple logistic regression (Table 3) it was also found that history of depression, unplanned pregnancy, being housewife (a probable indicator of low education and poverty) and having 3 or more children were variables with significant relation to ante partum depression.

Past history of depression has been consistently found to be one of the strong risk factors for PPD^25^,^32^,^33^ and women with a history of mood disorder have a 30% chance of developing PPD^34^ while one study reported that 78.3% of the women with PPD had a past and/or family history of depression.^35^

## Conclusions

Despite multiple psychosocial risk factors proposed as postpartum depression risk factor in previous studies, table 4 reveals that on a multiple logistic regression analysis two main risk factors of post partum depression in this cohort study were previous history of depression and depression during current pregnancy. This means that previous studied psychosocial risk factors of postpartum depression may be general risk factors for depression in any periods of a woman’s life.

The present study confirmed the importance of these two variables (previous history of depression and symptoms of depression during pregnancy) when screening women who may be at risk for PPD and highlighted the importance of assessing these two variables and as Austin mentioned “anxiety symptoms during pregnancy^16^ ” in order to facilitate timely identification of women at risk.

### 

#### Limitations

All eligible pregnant women were selected from whole area, for full coverage of different geographical and socio-economical participants.

Participants were all rural pregnant women in their third trimester of pregnancy who were recruited in this study and followed up from the beginning of the study to 6-8 weeks post-partum but not from urban area.

All of illiterate women (about 425 (%14) of 3000 subjects) were excluded from the study. It may violate the generality of the study.
